# Impact of screening on the prevalence and incidence of *Mycoplasma genitalium* and its macrolide resistance in men who have sex with men living in Australia: A mathematical model

**DOI:** 10.1016/j.eclinm.2021.100779

**Published:** 2021-03-03

**Authors:** Jason J. Ong, Luanqi Ruan, Aaron G. Lim, Catriona S. Bradshaw, David Taylor-Robinson, Magnus Unemo, Patrick J. Horner, Peter Vickerman, Lei Zhang

**Affiliations:** a Central Clinical School, Monash University, Victoria, Melbourne, Australia; b Faculty of Infectious and Tropical Diseases, London School of Hygiene and Tropical Medicine, London, United Kingdom; c China-Australia Joint Research Center for Infectious Diseases, School of Public Health, Xi'an Jiaotong University Health Science Center, Xi'an, Shaanxi, 710061, China; d Research Base of Key Laboratory of Surveillance and Early Warning on Infectious Disease, Shanghai Pudong New Area Center for Disease Control and Prevention, Shanghai, China; e Artificial Intelligence and Modelling in Epidemiology Program, Melbourne Sexual Health Centre, Alfred Health, Melbourne, Australia; f Population Health Sciences, Bristol Medical School, University of Bristol, Bristol, United Kingdom; g Faculty of Medicine, Imperial College London, London, United Kingdom; h WHO Collaborating Centre for Gonorrhoea and other STIs, Örebro University, Örebro, Sweden

## Abstract

**Background:**

*Mycoplasma genitalium* (MG) causes a sexually transmitted infection (STI) with a rising rate of antimicrobial resistance. Currently, guidelines do not recommend screening asymptomatic men who have sex with men (MSM). We developed a mathematical model of MG transmission to examine the impact of various screening strategies on the incidence and prevalence of MG among MSM attending a sexual health clinic.

**Methods:**

A compartmental mathematical model of MG transmission among MSM was constructed and calibrated using data from the Melbourne Sexual Health center, where resistance-guided therapy provides high treatment effectiveness (92–95%). The model stratified men by symptom status, sexual risk behaviours and whether or not they had MG with macrolide resistance. We simulated the impact on endemic steady-state MG prevalence and incidence of the following screening scenarios, namely screening: 1) no MSM; 2) only symptomatic MSM (the current recommendation); 3) all symptomatic and high-risk asymptomatic MSM; and 4) all MSM. Our base case analysis assumed a treatment effectiveness of 92–95% using resistance-guided therapy. We also examined the impact of treatment effectiveness (i.e. the proportion of detected MG that were cured) and screening coverage (i.e. testing rate) on MG prevalence.

**Findings:**

The model predicts that the overall endemic MG prevalence is 9.1% (95% CI: 7.9–10.0) in the current situation where screening is only offered to symptomatic MSM (base-case). This would increase to 11·4% (95% confidence intervals (CI): 10.2–13.7) if no MSM are offered screening, but would decrease to 7.3% (95% CI: 5.7–8.4) if all symptomatic and high-risk asymptomatic MSM were offered screening and 6.4% (95% CI: 4.7–7·7) if all MSM were offered screening. Increasing coverage of MSM screening strategies shows a similar effect on decreasing endemic MG incidence. When evaluating the simultaneous impact of treatment effectiveness and screening coverage, we found that offering screening to more MSM may reduce the overall prevalence but leads to a higher proportion of macrolide-resistant MG, particularly when using treatment regimens with lower effectiveness.

**Interpretation:**

Based on the available treatment options, offering screening for MG to other MSM (beyond the currently recommended group of symptomatic MSM) could slightly reduce the prevalence and incidence of MG. However, further increasing screening coverage must be weighed against the impact of lower treatment effectiveness (i.e. when not using resistance-guided therapy), increasing the selection of macrolide resistance, and other negative consequences related to AMR and management (e.g. unnecessary psychological morbidity from infections that do not need treatment).

**Research in context**

**Evidence before this study**

*Mycoplasma genitalium* (MG) is a sexually transmitted pathogen with rising antimicrobial resistance. There is no clear evidence regarding the optimal screening strategy to control MG. We conducted a scoping review for mathematical models evaluating *Mycoplasma genitalium* on 4th November 2020 in Medline, using the following key terms: ‘*Mycoplasma genitalium*’, ‘model’ and ‘screen* or test*’ and ‘men’. We found 217 papers: two publications had relevant data using dynamic transmission models to evaluate the impact of screening for MG among heterosexual populations and no publications evaluated screening among men who have sex with men (MSM).

**Added value of this study**

We explored various screening strategies for MG and found that including asymptomatic MSM in screening could slightly reduce the prevalence and incidence of MG. However, further increasing screening coverage must be weighed against the impact of lower treatment effectiveness (where resistance-guided therapy is not available), increasing the selection of macrolide resistance, and other negative consequences related to AMR and management (e.g. unnecessary psychological morbidity from infections that do not need treatment).

**Implications of all the available evidence**

We provide evidence to support the current expert opinion in MG guidelines to discourage screening for MG among asymptomatic MSM.

Alt-text: Unlabelled box

## 1 Introduction

*Mycoplasma genitalium* (MG) is a bacterium, first discovered and named in the 1980s, that causes sexually transmitted infection (STI) and disease in the lower and upper reproductive tract of women, and non-gonococcal urethritis in men. [1], [2], [3] Reported overall MG prevalence ranges from 1.3% (in countries with a high/very high Human Development Index (HDI)) to 3.9% (in countries with a lower HDI). [4] In many countries, MG is the second most common bacterial STI, after *Chlamydia trachomatis*. A major challenge is the emergence of antimicrobial resistance (AMR) in MG, notably the rapid rise of macrolide-resistant MG. [5] The treatment efficacy of azithromycin continues to decrease over time. [6] Also, the emergence of resistance to the second-line antibiotic, the fluoroquinolone moxifloxacin, is increasingly reported [7] and this has posed the question of whether infection with MG could become untreatable in the near future. [8]

A critical strategy underpinning control of non-vaccine preventable infectious diseases is to screen and treat the pathogen to reduce the duration of infectiousness and, thus, the likelihood of ongoing transmission in the population. This is particularly important for infections like that caused by MG where the majority of those infected presenting to sexual health clinics are asymptomatic, i.e. 93% of anorectal infections and 79% of urethral infections in MSM. [9] To date, there is limited guidance on who should be screened, despite increasing use of nucleic acid amplification tests for the diagnosis of MG in clinical settings (including molecular tests to allow detection of MG-AMR). The European guidelines recommend testing for MG in individuals with urethritis, cervicitis, intermenstrual or post-coital bleeding, acute pelvic pain or pelvic inflammatory disease and acute epididymo-orchitis (in men below 50 years old). [10] The Australian and British national guidelines are similar to these in that there is no recommendation for screening asymptomatic individuals, even if they are from high-risk populations [11,12] due to lack of a sufficiently effective treatment and fears of rising AMR. [8] However, it is not clear how ongoing transmission in this large reservoir of MG-infected but asymptomatic individuals is contributing to the prevalence and incidence of MG and, particularly, its effect on AMR. Critically, it is unclear what type of screening strategies could reduce the prevalence and incidence of MG infections and macrolide-resistant MG strains, and what level of coverage might be needed to achieve this.

Mathematical models have been successfully used to inform public health policies regarding interventions to control a range of STIs. [13,14] The aim of our study was to evaluate the impact of various screening scenarios (universal vs. targeted screening) on MG prevalence and incidence (including macrolide resistance) in MSM, based on data from the Melbourne Sexual Health center (MSHC), Australia.

## 2 Methods

### 2.1 Model description

We developed a compartmental model to describe the transmission of MG among MSM. The model stratifies the population by symptom status (symptomatic or asymptomatic), sexual risk behaviours (low- or high-risk) and whether the infection was with wild-type or macrolide-resistant MG. Symptomatic men were defined as those with either urethritis or proctitis. We defined high-risk men as those with more than ten (anal) sexual partners in the preceding six months, according to the guidelines of the Sexually Transmissible Infections in Gay Men Action Group (STIGMA). [15] Approximately 18% of men visiting the MSHC are classified as high-risk. [16]

**Fig. 1:**
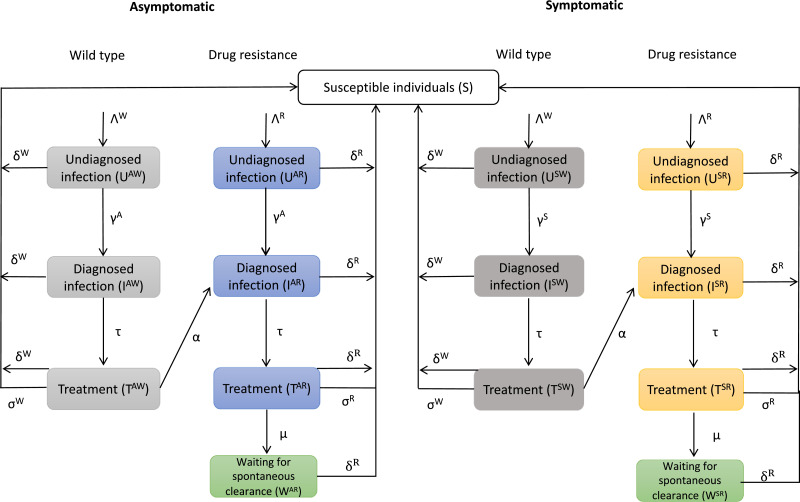
Model structure of *Mycoplasma genitalium* in low-risk men who have sex with men. Λ refers to the rate of being infected with either wild-type (Λ^W^) or macrolide-resistant *Mycoplasma genitalium* (Λ^R^). γ refers to the rate of diagnosis for asymptomatic (γ^A^) or symptomatic men (γ^S^). τ refers to the rate of being treated for MG. δ refers to the rate of spontaneous clearance for wild-type (δ_W_) or macrolide-resistant MG (δ_R_). σ refers to the rate of cure with antibiotics for wild-type (σ^W^) or macrolide-resistant MG (σ^R^). μ refers to the rate of developing MG that is incurable with current antibiotics (azithromycin, sitafloxacin/moxifloxacin, pristinamycin, minocycline). A similar diagram exists for high-risk men, with sexual mixing occurring between low- and high-risk men.

Fig. 1 illustrates the compartmental structure of the model for symptomatic and asymptomatic men. We assume there are no transitions between low- and high-risk men, but sexual mixing can occur between the two populations; we used a previously published matrix to define sexual mixing between these groups. [16] The matrix accounted for heterogeneity in sexual mixing of high- and low-risk groups and was informed by data from the MSHC. MSHC is a public sexual health clinic in Melbourne, Australia with nearly 60,000 consultations a year of which a third are MSM. On attendance, clients fill out a computer assisted survey interview which includes details on their sexual behaviours. More details on the model are provided in Appendix A.

At any specific time, a man is in one of the compartments and can move between compartments (following the direction of the arrows) over time as determined by the transition rates. Susceptible men (S) can become infected (and undiagnosed) by another MSM with either wild-type or macrolide-resistant type MG infection and are initially undiagnosed. As in other models, we assume the transmission rate is independent of symptom status and the type of MG strain. [17] A proportion of infected men will be tested and diagnosed with MG, whereupon antibiotics are provided for treatment. Consistent with our current clinic management and based on recommended clinical practice in Australia, we assume men are tested with a macrolide-resistance assay that provides information on MG infection as well as distinguishing those with macrolide-resistant MG. Only a proportion of men receiving antibiotics will be cured depending on the AMR profile of MG. The estimate of effectiveness is based on data from MSHC: cases are given doxycycline 100 mg bd 7 days followed either by azithromycin (1 g, then 500 mg daily for 3 days) for macrolide-susceptible cases, or moxifloxacin (400 mg daily 7 days) or sitafloxacin (100 mg bd 7 days) for macrolide-resistant cases. [18] We accounted for the possibility of *de novo* emergence of resistance during therapy with a macrolide. [19] Our model allows MG, whether wild-type or macrolide-resistant, to spontaneously clear in the absence of detection and treatment. [20] Men become susceptible to infection again after successful treatment or spontaneous clearance with no development of short- or long-term immunity to MG, as evidenced by reinfection rates. [20] If a man with treated MG develops macrolide resistance, we moved this man to the compartment of diagnosed resistant MG because it is our clinical practice to do a test of cure for every man diagnosed with MG. Men with resistant MG that is untreatable with current antibiotics (including use of pristinamycin or minocycline) enter a compartment where they cannot be treated (‘waiting for spontaneous clearance’ box in Fig. 1), and from which they eventually spontaneously clear their infection, as observed clinically. Our model investigates the equilibrium level of the prevalence/incidence of MG by assuming a closed population without the incorporation of births or deaths.

### 2.2 Model parameters and calibration (Table 1)

To parameterize our model, we used clinical data from MSM attending the MSHC, and where necessary, other parameter estimates were derived from published literature, as shown in Table 1. Authors JO and LR had access to the data and validated the input data sources. To explore the large parameter space for calibration, we randomly generated 1 million parameter sets from the known parameter uncertainty ranges (ranges shown in Table 1) based on Latin Hypercube sampling. For each set of the parameters, we simulated the model to obtain the corresponding equilibrium prevalence, which was subsequently compared with the actual prevalence data from MHSC. The goodness-of-fit was determined as the root mean squared error between the simulated equilibrium prevalence and the empirical prevalence. We then ranked the simulations by their goodness-of-fit and used the parameter sets associated with the top 1% (10,000 simulations) to define a reduced parameter space. From these, we randomly sampled parameter sets for further optimization. For each parameter set, the model was calibrated through using the MATLAB routine *fsolve* (based on a ‘trust-region dogleg’ algorithm) to minimize the error between the model and baseline prevalence data from MHSC. The calibration was considered successful when a stable baseline MG prevalence at equilibrium was reached as observed in MSHC in 2018 among MSM screened for MG regardless of symptoms status: 3.8% (95% CI: 2.5–5.1) for wild-type MG in high-risk men; 0.9% (95% CI: 0.6–1.2) for wild-type MG in low-risk men; 16.7% (95% CI: 14.1–19.3) for macrolide-resistant MG in high-risk men; and 5.7% (95% CI: 4.9–6.5) for macrolide-resistant MG in low-risk men. [21] We rejected any parameter set that could not generate modelled prevalences within the estimated 95% confidence intervals of these observed prevalences. This optimization process was repeated until a total of 200 successful calibrations were reached. These parameter sets were used as the baseline model fits for running simulations under the intervention scenarios.

**Table 1:** Model parameters based on previous studies and model calibration for men who have sex with men.

Parameter	Description	Point Estimate	Sampled range used in model fitting#	Final model estimates (mean, 95% CrI)	Reference
C_H_	Average consistency of condom use in the last anal sex act (%) for high-risk men	40	27–53	42 (37–48)	MSHC (not shown)
C_L_	Average consistency of condom use in last anal sex act (%) for low-risk men	60	40–80	60 (53–68)	MSHC (not shown)
f_H_	Average frequency of anal sex in the past week among high-risk men	3.5	2.3–15	6.7 (5.8–8.1)	MSHC (not shown)
f_L_	Average frequency of anal sex in the past week among low-risk men	1.1	0.7–5·0	3·1 (2·5–3·5)	MSHC (not shown)
β	Per-act transmission of MG per unprotected sex (%)	3	2–50	10·0 (8·6–13·2)	Assumption based on *Chlamydia trachomatis* (Tu, 2018)[22]
ε	% condom efficacy in preventing transmission per anal sex	88	80–95	87 (85–90)	(Warner, 2004)[23]
superscript ^A^ or ^S^	% men who were asymptomatic	92	61–100	90 (87–93)	MSHC (not shown)
$\gamma^{A}$	Testing rate per week for asymptomatic men	0·0005	0–0·001	0·0005 (0·0003–0·0007)	MSHC (not shown)
$\gamma^{S}$	Testing rate per week for symptomatic men	0·023	0·013–0·130	0·06 (0·03–0·09)	MSHC (not shown)
τ	% men who receive treatment if diagnosed	77	72–82	78 (76–81)	(Ong, 2018)[3]
$\sigma^{W}$	% successful treatment for wild-type MG (per week)	95	80–100	89 (85–93)	(Read, 2019)[18]
$\sigma^{R}$	% successful treatment for resistant MG (per week)	92	80–100	86 (83–89)	(Read, 2019)[18]
α	% Treated wild-type MG developing into resistant-MG	12	0–20	6 (4–7)	(Horner, 2018)[19]
μ	rate of macrolide-resistant MG that failed treatment with antibiotics per week	0·05	0–0·20	0·09 (0·02–0·20)	(Read, 2019)[18]
$\delta^{W}$	spontaneous clearance rate of wild-type MG per week	0·02	0·01–0·2	0·13 (0·10–0·16)	(Smieszek, 2016)[24]
$\delta^{R}$	spontaneous clearance rate of resistant MG per week	0·02	0·01–0·2	0·13 (0·10–0·16)	(Smieszek, 2016)[24]

CrI = credible intervals; MSHC = data directly from Melbourne Sexual Health center, 2018 (not shown).

# Fitting constraints are based on 95% confidence intervals or what is clinically plausible.

### 2.3 Model and statistical analysis

The primary outcome measures were the impact of different MG screening strategies on the prevalence and incidence of MG (wild-type and macrolide-resistant) for four groups of MSM according to their risk (high or low) and symptom status (symptomatic or asymptomatic). The model of the current recommendation (scenario 2) was offering MG screening to MSM who had symptoms (i.e. standard practice at the MSHC since 2016). We evaluated the impact of different screening scenarios by keeping all parameters shown in Table 1 constant, except for those who were offered MG screening: Scenario 1) no MSM screening (i.e. the counterfactual); Scenario 2) screen only symptomatic MSM (i.e. the current recommendation, for which weekly screening rate is 0·023 for symptomatic MSM; and a smaller weekly rate (0·0005) of asymptomatic men who might be inadvertently screened as observed in our clinic); Scenario 3) screen all symptomatic (low and high-risk) and high-risk asymptomatic MSM (i.e. assuming weekly screening rates is 0·023 for these three groups); and Scenario 4) all MSM (i.e. assuming weekly screening rate is 0·023 for all MSM). A weekly testing rate of 0·023 corresponds to ~70% probability of being tested over a year, using the transformation described here. [25] In scenario 3, we tested whether offering MG screening (0.023 weekly screening rate) to high-risk asymptomatic men (who have a higher MG prevalence) would affect the dynamics of macrolide-resistant strains, as is proposed for *Neisseria gonorrhoeae.* [26]

We created a heat map to demonstrate the simultaneous impact on MG prevalence of varying treatment effectiveness and screening rate for all symptomatic men (i.e. current practice, Scenario 2). We present the impact on the prevalence of wild-type MG and macrolide-resistant MG, disaggregated by risk behavior. We also conducted univariate sensitivity analyses using the 95% credible intervals of all the model inputs as the lower and upper bounds, using the current recommendation scenario (i.e. screening symptomatic men only) and present the top eight most influential parameters using a tornado plot.

As this was a modeling study, ethics approval was not required.

## 3 Role of the funding source

None.

## 4 Results

A total of 200 simulations fitted to baseline data were used for the final analysis. The model was used to project the prevalence and incidence of wild-type and macrolide-resistant MG for high- and low-risk MSM according to the different screening scenarios, as shown in Table 2. For the current practice of screening (scenario 2: mainly screening symptomatic MSM only), the model was well-calibrated to the observed prevalence of MG among high- and low-risk MSM seen at the MSHC. If no screening was conducted, the overall prevalence and incidence of MG would increase, whereas it decreases in all scenarios where there was greater screening coverage. The model predicts that the overall MG prevalence will be 11.4% (95% confidence intervals (CI): 10.2–13.7) if no MSM are offered screening (Scenario 1); 9.1% (95% CI: 7.9–10.0) if screening is mainly offered to symptomatic MSM (Scenario 2, the current practice scenario); 7.3% (95% CI: 5.7–8.4) if offered to symptomatic and high-risk asymptomatic MSM (Scenario 3); and 6·4% (95% CI: 4.7–7.7) if offered to all MSM (Scenario 4). The model predicts that the overall incidence of MG among MSM will be 34.3 per 100 person-years (95% CI: 28.1–41.7) if no MSM are offered MG screening (Scenario 1); 29.5 per 100 person-years (95% CI: 23.1–36.7) if screening is offered to only symptomatic MSM (Scenario 2); 24.4 per 100 person-years (95% CI: 16.7–33.4) if offered to symptomatic and high-risk asymptomatic MSM (Scenario 3); and 22.9 per 100 person-years (95% CI: 15.0–32.2) if offered to all MSM (Scenario 4).

**Table 2:** Steady state prevalence and incidence of wild-type and macrolide-resistant *Mycoplasma genitalium* according to screening scenarios in Australian men who have sex with men.

	Wild type		Macrolide-resistant		
Screening	High risk*	Low risk	High risk*	Low risk	
	*Prevalence*% (95% CrI)	*Prevalence*% (95% CrI)	*Prevalence*% (95% CrI)	*Prevalence*% (95% CrI)	*Overall prevalence*% (95% CrI)
Scenario 1: No one is screened	11·5 (4·2–23·2)	3·9 (1·2–8·8)	13·4 (4·8–21·1)	4·6 (1·8–7·0)	11·4 (10·2–13·7)
Scenario 2: Symptomatic MSM only (current recommendation)	2·9 (1·8–4·8)	0·9 (0·6–1·5)	17·6 (14·6–19·5)	5·6 (5·1–6·5)	9·1 (7·9–10·0)
Scenario 3: Symptomatic and high-risk asymptomatic	0·5 (0·0–1·2)	0·2 (0·0–0·4)	15·3 (12·0–18·4)	5·2 (4·1–6·3)	7·3 (5·7–8·4)
Scenario 4: All men	0·3 (0·0–0·9)	0·1 (0·0–0·3)	14·6 (10·8–18·0)	4·4 (3·2–5·4)	6·4 (4·7–7·7)
	*Incidence* per 100 PY	*Incidence* per 100 PY	*Incidence* per 100 PY	*Incidence* per 100 PY	*Overall incidence* per 100 PY
Scenario 1: No one is screened	15·2 (5·8–29·4)	18·0 (5·7–34·5)	17·4 (5·4–26·1)	20·5 (7·2–31·1)	34·3 (28·1–41·7)
Scenario 2: Symptomatic MSM only (current recommendation)	4·4 (2·4–8·8)	5·0 (2·9–10·1)	23·5 (18·3–28·7)	26·4 (21·0–30·4)	29·5 (23·1–36·7)
Scenario 3: Symptomatic and high-risk asymptomatic	0·8 (0·1–1·8)	0·8 (0·1–2·1)	22·1 (15·5–29·4)	24·2 (16·5–32·8)	24·4 (16·7–33·4)
Scenario 4: All men	0·5 (0·0–1·5)	0·6 (0·0–1·7)	21·2 (14·1–29·1)	22·7 (14·8–31·9)	22·9 (15·0–32·2)

⁎ high-risk men are those who report more than 10 sexual partners in the last six months, and make up 18% of the MSM population at Melbourne Sexual Health center 95% CrI = 95% credible interval; PY = person-years.

**Fig. 2:**
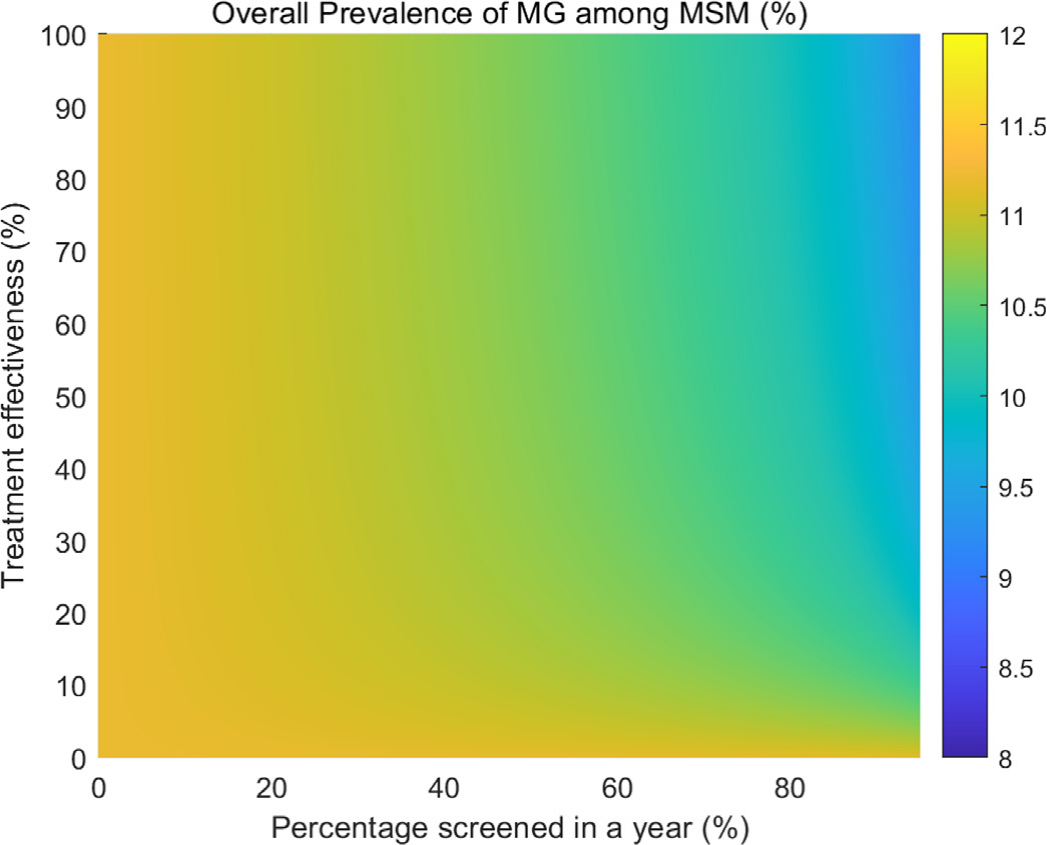
Heatmap of the impact of treatment effectiveness and screening coverage on the overall *Mycoplasma genitalium* prevalence for Australian men who have sex with men.

**Fig. 3:**
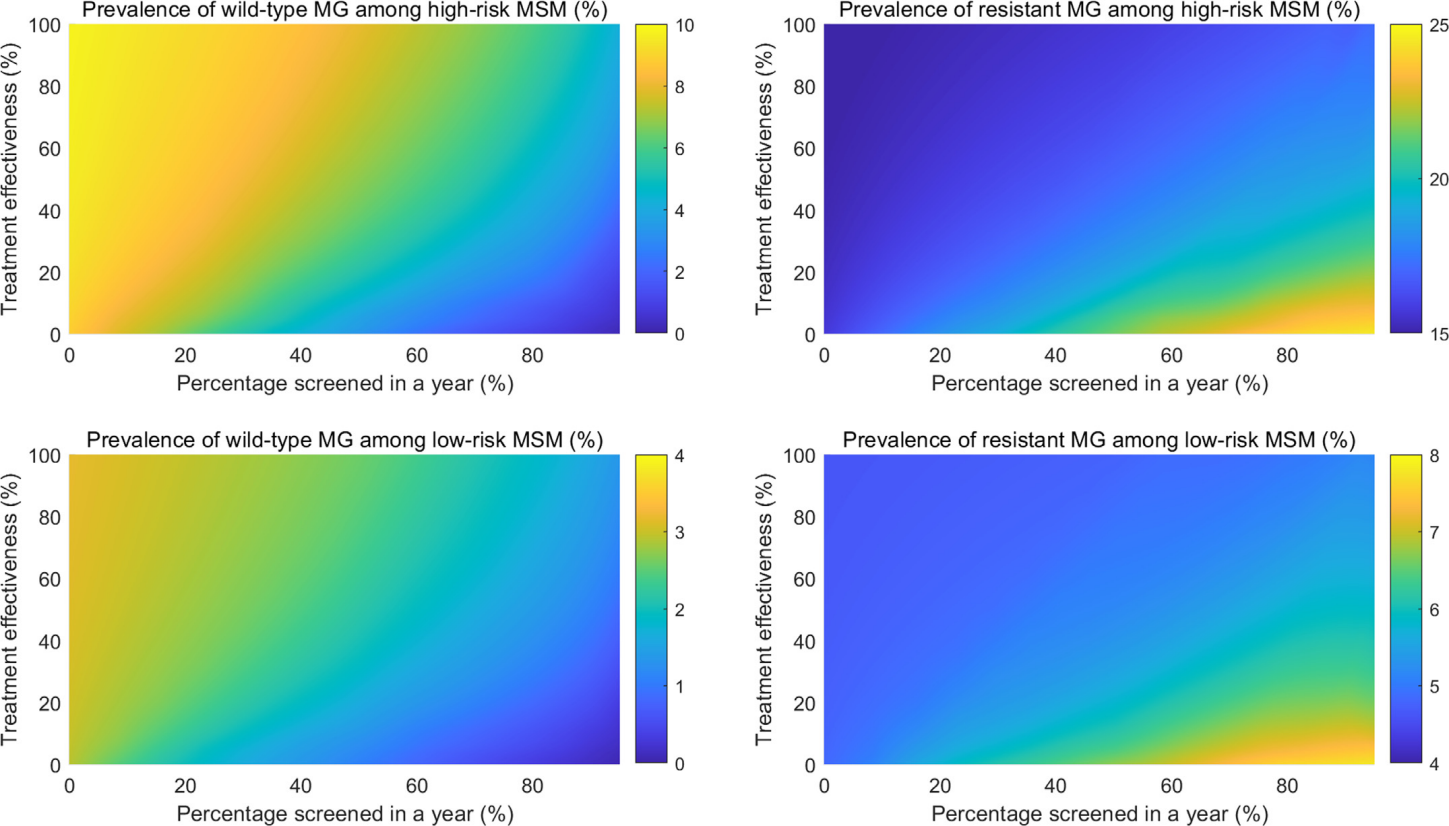
Heatmap of the impact of treatment effectiveness and screening coverage on wild-type and macrolide-resistant *Mycoplasma genitalium* prevalence for low- and high-risk Australian men who have sex with men.

We explored the simultaneous impact of varying treatment effectiveness and varying screening coverage among symptomatic MSM (Scenario 2: the current practice scenario) on the overall MG prevalence for all men. Fig. 2 demonstrates that the overall MG prevalence will decrease with higher treatment effectiveness combined with higher percentage of men screened in a year. To further understand the distribution of wild-type and macrolide-resistant MG among low- and high-risk men, Fig. 3 shows that with a higher screening coverage, there is a higher prevalence of macrolide-resistant MG in both low- and high-risk men, especially when treatment effectiveness decreases.

**Fig. 4:**
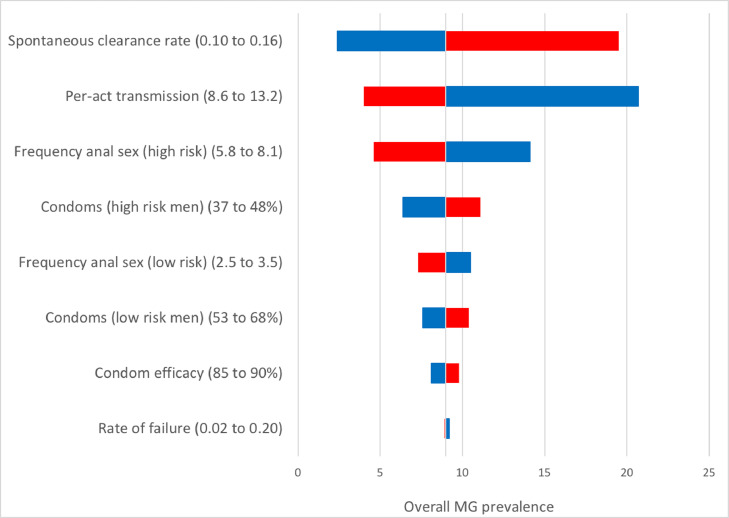
Univariate sensitivity analysis of *Mycoplasma genitalium* prevalence for Australian men who have sex with men Red bars correspond to the effect of the low value in the sensitivity analysis, and blue bars correspond to the high value in the sensitivity analysis.

Fig. 4 is the tornado plot to summarize the univariate sensitivity analyses. This figure demonstrates the impact on the overall MG prevalence of the uncertainty of the input values by changing one parameter at a time over a range of plausible values. This identifies how influential certain parameters are, and thus the importance to obtain accurate data regarding these. The overall MG prevalence is most sensitive to the assumptions regarding the weekly spontaneous clearance rate, the per-act transmission (%) of MG, and the frequency of anal sex among high-risk MSM (within the past week). We present the effect of changing the most sensitive parameter (i.e. spontaneous clearance rate) on prevalence in Appendix 2. This demonstrates the effect of changing this parameter for scenarios presented in Table 2, and underscores the importance of accurately measuring these parameters.

## 5 Discussion

Current international and national guidelines discourage screening of asymptomatic MSM for MG due to limited knowledge regarding the natural history of MG infections in MSM, rising AMR, and increasing complexities and costs associated with treatment. [10], [11], [12] However, these recommendations are based on expert opinion. Our dynamic transmission model of MG calibrated to data from MSM in Melbourne provides further evidence to support these recommendations and their continued use. We show that expanding screening beyond symptomatic MSM using highly effective treatment regimens might reduce MG prevalence and incidence overall, but is likely to increase the prevalence and incidence of macrolide-resistant MG. When resistance-guided therapy is not available, we expect reduced treatment effectiveness due to rising macrolide resistance in MG; [27] in this scenario, we demonstrate that increasing screening coverage using treatment regimens with lower treatment effectiveness is more likely to increase macrolide-resistant MG prevalence. Our model uses a high-estimate of effectiveness because we use resistance-guided therapy in our setting, but it is likely that effectiveness is much lower in the absence of resistance-guided therapy. [8]

To our knowledge, this is the first mathematical modeling study exploring the likely impact of MG screening strategies for MSM. There are two published transmission dynamic models in which the impact of screening for MG has been evaluated for heterosexual populations. [28,29] The model using data from heterosexual populations in the UK suggests that MG testing should occur for both asymptomatic and symptomatic women to reduce the risk of pelvic inflammatory disease. [28] Compared to women, the health consequences of MG among MSM appear to be minimal as many men with MG do not experience symptoms, and serious sequelae in MSM have not been reported. [21] The other model suggests that using antibiotic-resistance guided therapy could increase the proportion with macrolide-susceptible MG among symptomatic heterosexual populations, but the impact of different screening coverages or the effect of screening asymptomatic individuals was not explored. [29] The findings from these two models, together with those from our model, suggest that there is value in further exploring the cost-effectiveness of various strategies to control MG as there are significant resource implications for implementing MG screening.

There are important considerations when recommending population screening for MG. While international STI guidelines recommend screening asymptomatic individuals for STIs such as chlamydial and gonococcal infections, this is not so for MG. In this case, there are concerns about lack of effective treatment, rising AMR (in MG and other STIs), increased costs and adverse effects of treatment, lack of knowledge about the natural history of MG in MSM and unnecessary psychological morbidity among screened MSM. For MSM, MG is not associated with the reproductive morbidity seen in women. [12] Furthermore, antimicrobial therapy is not without risk, with potential for uncommon but serious side effects [30], and overuse is a driver for AMR. [31] Our finding that increasing the proportion of MSM screened would also increase the proportion of macrolide-resistant MG is due to MG's propensity to develop resistance when exposed to antibiotics as reported in empirical data. [19] This is also consistent with another model using data from France, Denmark and Sweden which revealed that blind treatment of urethritis with macrolides contributed to the spread of macrolide-resistant MG. [17] We are planning to examine the trade-offs between reducing overall MG prevalence and the impact of increased proportion of macrolide-resistant MG using a cost-effectiveness analysis of MG screening in a future study.

The main strength of this research is that we developed a model structure that captured the key features of MG infection using currently available, real-world data. For parameters that were not available, we derived estimates by fitting the model to data from Australian MSM. Our research has several limitations. We calibrated the model to MG prevalence among MSM attending the MSHC in Australia. This was a pragmatic approach due to a lack of robust estimates of MG epidemiology in the community. Our study may thus over- or under-estimate the impact of screening if the MG epidemic in the wider MSM community substantially differs from that of MSM attending the MSHC. As more data on MG become increasingly available, particularly from other countries and community-based estimates, our model can be re-calibrated to examine its external validity. Our model focused on MSM; future models that account for the heterosexual and bisexual transmission of MG, and bridging between the populations, would also be useful to understand the potential impact of screening in the wider population, particularly where MG may cause reproductive morbidity in heterosexual women. There is potential for model misspecification as there is limited data on the natural history of MG among MSM. We reported the impact of uncertain model input parameters in our sensitivity analyses to demonstrate the potential effects of these unknown parameters. In addition, our model was calibrated to MG prevalence among symptomatic and asymptomatic men from a clinical study in 2018 [21]. Future studies to measure MG prevalence among asymptomatic men will be helpful to determine if steady-state was reached. Finally, we reported our findings using steady-state prevalence and incidence. This assumes that the number of people with MG at any time period is balanced between new infections that are occurring and previously infections that have been treated or that have naturally cleared under the scenario of interest. This provides decision-makers with a comparable endpoint, as our models do not determine how long it will take to reach these steady-states.

We explored various screening strategies for MG and found that including asymptomatic MSM in screening could slightly reduce the prevalence or incidence of MG. However, further increasing screening coverage must be weighed against the impact of lower treatment effectiveness (where resistance-guided therapy is not available), increasing the selection of macrolide resistance, and other negative consequences related to AMR and management (e.g. unnecessary psychological morbidity from infections that do not need treatment).

## Data sharing

The model code (in Matlab) is available on request to the corresponding author.

## Contributors

JJO and LZ designed the research study. JJO, LR, and LZ created the model. AGL and PV critiqued the model and subsequent analyses. JJO, LR, AGL, CB, DTR, MU, pH, PV, LZ analyzed the data. JJO wrote the paper and all authors have read and approved the final manuscript.

## Declaration of Competing Interest

None to declare.

## Appendix Supplementary materials

Image, application 1
