# Supplementary material for: Impact of screening on the prevalence and incidence of Mycoplasma genitalium and its macrolide resistance in men who have sex with men living in Australia: A mathematical model
Source: eClinicalMedicine. 2021 Mar 3;33:100779. doi: 10.1016/j.eclinm.2021.100779 (PMC8020166; doi:10.1016/j.eclinm.2021.100779)
Supplement: Supplementary file 1 [file mmc1.docx]

**Supplementary Figure 1**


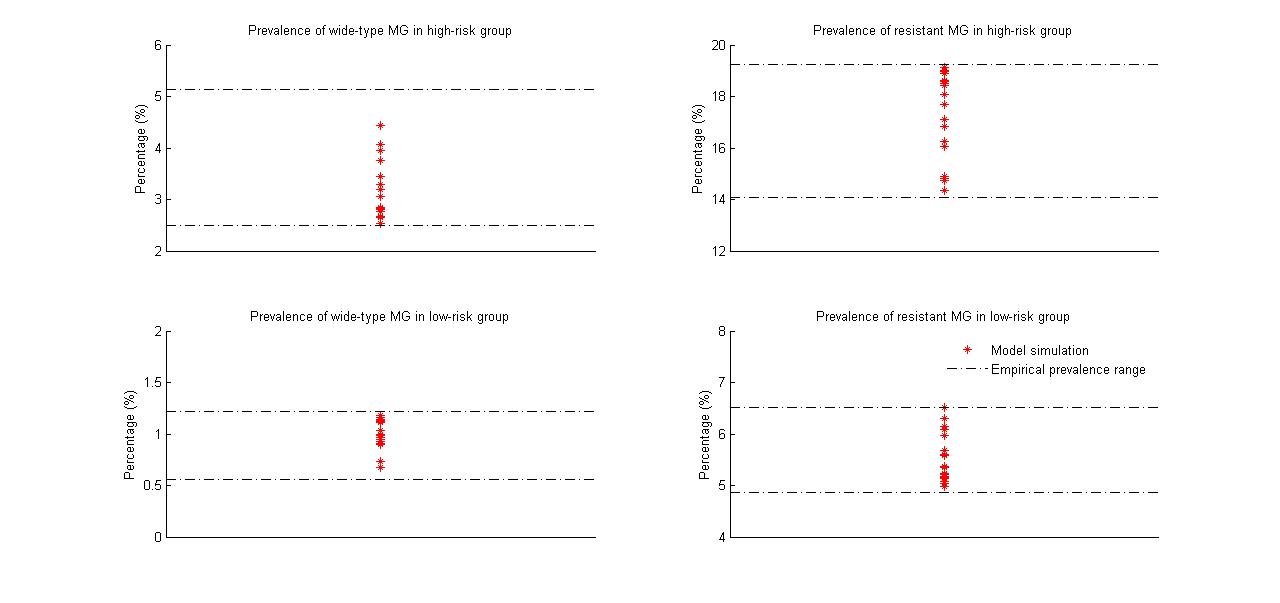


**Appendix 2**

This is a sensitivity analysis to demonstrate the impact of changing the parameter of the natural clearance rate of *Mycoplasma genitalium.*

**Current model**

|  | Wild type | | Macrolide-resistant | |  |
| --- | --- | --- | --- | --- | --- |
| Screening | High risk* | Low risk | High risk* | Low risk |  |
|  | *Prevalence*  % (95% CrI) | *Prevalence*  % (95% CrI) | *Prevalence*  % (95% CrI) | *Prevalence*  % (95% CrI) | *Overall prevalence*  % (95% CrI) |
| Scenario 1: No one is screened | 11·5 (4·2-23·2) | 3·9 (1·2-8·8) | 13·4 (4·8-21·1) | 4·6 (1·8-7·0) | 11·4 (10·2-13·7) |
| Scenario 2: Symptomatic MSM only (current recommendation) | 2·9 (1·8-4·8) | 0·9 (0·6-1·5) | 17·6 (14·6-19·5) | 5·6 (5·1-6·5) | 9·1 (7·9-10·0) |
| Scenario 3: Symptomatic and high-risk asymptomatic | 0·5 (0·0-1·2) | 0·2 (0·0-0·4) | 15·3 (12·0-18·4) | 5·2 (4·1-6·3) | 7·3 (5·7-8·4) |
| Scenario 4: All men | 0·3 (0·0-0·9) | 0·1 (0·0-0·3) | 14·6 (10·8-18·0) | 4·4 (3·2-5·4) | 6·4 (4·7-7·7) |
|  | *Incidence*  per 100 PY | *Incidence*  per 100 PY | *Incidence*  per 100 PY | *Incidence*  per 100 PY | *Overall incidence*  per 100 PY |
| Scenario 1: No one is screened | 15·2 (5·8-29·4) | 18·0 (5·7-34·5) | 17·4 (5·4-26·1) | 20·5 (7·2-31·1) | 34·3 (28·1-41·7) |
| Scenario 2: Symptomatic MSM only (current recommendation) | 4·4 (2·4-8·8) | 5·0 (2·9-10·1) | 23·5 (18·3-28·7) | 26·4 (21·0-30·4) | 29·5 (23·1-36·7) |
| Scenario 3: Symptomatic and high-risk asymptomatic | 0·8 (0·1-1·8) | 0·8 (0·1-2·1) | 22·1 (15·5-29·4) | 24·2 (16·5-32·8) | 24·4 (16·7-33·4) |
| Scenario 4: All men | 0·5 (0·0-1·5) | 0·6 (0·0-1·7) | 21·2 (14·1-29·1) | 22·7 (14·8-31·9) | 22·9 (15·0-32·2) |

**Natural clearance is 20% lower**

|  | Wild type | | Macrolide-resistant | |  |
| --- | --- | --- | --- | --- | --- |
| Screening | High risk* | Low risk | High risk* | Low risk |  |
|  | *Prevalence*  % (95% CrI) | *Prevalence*  % (95% CrI) | *Prevalence*  % (95% CrI) | *Prevalence*  % (95% CrI) | *Overall prevalence*  % (95% CrI) |
| Scenario 1: No one is screened | 16·0 (6·9-30·1) | 6·6 (2·3-13·9) | 24·9 (12·4-34·3) | 10·2 (5·5-14·3) | 21·4 (12·8-32·1) |
| Scenario 2: Symptomatic MSM only (current recommendation) | 4·1 (2·6-5·2) | 1·6 (1·1-2·1) | 32·7 (29·8-34·6) | 12·5 (10·7-14·4) | 18·2 (16·9-19·9) |
| Scenario 3: Symptomatic and high-risk asymptomatic | 0·7 (0·1-2·3) | 0·3 (0-1·0) | 31·0 (27·5-34·2) | 12·9 (10·1-14·7) | 16·5 (14·4-18·3) |
| Scenario 4: All men | 0·4 (0-1·8) | 0·2 (0-0·7) | 30·3 (26·9-33·6) | 10·8 (8·3-12·3) | 14·5 (12·5-16·2) |
|  | *Incidence*  per 100 PY | *Incidence*  per 100 PY | *Incidence*  per 100 PY | *Incidence*  per 100 PY | *Overall incidence*  per 100 PY |
| Scenario 1: No one is screened | 21·1 (18·8-23·8) | 16·9 (7·6-32·0) | 23·4 (9·0-43·0) | 24·8 (10·9-32·6) | 47·3 (31·1-63·7) |
| Scenario 2: Symptomatic MSM only (current recommendation) | 4·9 (3·2-7·8) | 6·7 (4·2-10·7) | 33·1 (27·8-39·1) | 42·5 (35·4-49·8) | 44·7 (37·3-53·3) |
| Scenario 3: Symptomatic and high-risk asymptomatic | 0·9 (0·1-3·0) | 1·2 (0·1-4·0) | 34·0 (27·6-42·2) | 42·1 (33·4-52·3) | 41·4 (32·8-51·4) |
| Scenario 4: All men | 0·6 (0-2·4) | 0·8 (0-3·1) | 33·3 (26·7-41·9) | 40·5 (31·5-51·4) | 39·8 (31·0-50·2) |

**Natural clearance is 20% higher**

|  | Wild type | | Macrolide-resistant | |  |
| --- | --- | --- | --- | --- | --- |
| Screening | High risk* | Low risk | High risk* | Low risk |  |
|  | *Prevalence*  % (95% CrI) | *Prevalence*  % (95% CrI) | *Prevalence*  % (95% CrI) | *Prevalence*  % (95% CrI) | *Overall prevalence*  % (95% CrI) |
| Scenario 1: No one is screened | 4·7 (1·5-11·3) | 1·3 (0·4-3·6) | 4·0 (1·2-7·7) | 1·2 (0·4-1·9) | 3·6 (1·8-5·5) |
| Scenario 2: Symptomatic MSM only (current recommendation) | 0·8 (0-1·7) | 0·2 (0-0·5) | 3·4 (0-5·6) | 0·9 (0-1·3) | 1·7 (0-2·6) |
| Scenario 3: Symptomatic and high-risk asymptomatic | 0·1 (0-0·2) | 0·01 (0-0·06) | 1·0 (0-3·1) | 0·3 (0-0·8) | 0·4 (0-1·3) |
| Scenario 4: All men | 0·03 (0-0·13) | 0·01 (0-0·03) | 0·8 (0-2·6) | 0·2 (0-0·6) | 0·2 (0-1·0) |
|  | *Incidence*  per 100 PY | *Incidence*  per 100 PY | *Incidence*  per 100 PY | *Incidence*  per 100 PY | *Overall incidence*  per 100 PY |
| Scenario 1: No one is screened | 7·7 (2·5-15·4) | 7·9 (2·1-16·5) | 6·6 (1·6-12·5) | 6·7 (1·9-11·3) | 14·1 (8·2-18·4) |
| Scenario 2: Symptomatic MSM only (current recommendation) | 1·5 (0-3·8) | 1·5 (0-3·8) | 6·1 (0·1-10·1) | 5·8 (0·1-9·3) | 7·2 (0·1-11·9) |
| Scenario 3: Symptomatic and high-risk asymptomatic | 0·1 (0-0·5) | 0·1 (0-0·5) | 2·0 (0-6·2) | 1·8 (0-5·5) | 1·9 (0-6·0) |
| Scenario 4: All men | 0·1 (0-0·3) | 0·1 (0-0·3) | 1·7 (0-5·2) | 1·5 (0-4·6) | 1·6 (0-4·9) |
